# Using a multistate occupancy approach to determine molecular diagnostic accuracy and factors affecting avian haemosporidian infections

**DOI:** 10.1038/s41598-020-65523-x

**Published:** 2020-05-21

**Authors:** Raquel A. Rodrigues, Rodrigo L. Massara, Larissa L. Bailey, Mauro Pichorim, Patrícia A. Moreira, Érika M. Braga

**Affiliations:** 1 Departamento de Parasitologia, Instituto de Ciências Biológicas, Universidade Federal de Minas Gerais, Belo Horizonte, MG Brazil; 2 Departamento de Genética, Ecologia e Evolução, Instituto de Ciências Biológicas, Universidade Federal de Minas Gerais, Belo Horizonte, MG Brazil; 3 Instituto SerraDiCal de Pesquisa e Conservação, Belo Horizonte, MG Brazil; 4 Department of Fish, Wildlife and Conservation Biology and Graduate Degree Program in Ecology, Colorado State Univ, Fort Collins, CO USA; 5 Departamento de Botânica e Zoologia, Universidade Federal do Rio Grande do Norte, Natal, Rio Grande do Norte Brazil; 6 Departamento de Biodiversidade, Evolução e Meio Ambiente, Instituto de Ciências Exatas e Biológicas, Universidade Federal de Ouro Preto, Ouro Preto, MG Brazil

**Keywords:** Conservation biology, Ecological modelling, Population dynamics

## Abstract

The use of a sensitive and accurate parasite detection methodology is crucial in studies exploring prevalence of parasites in host populations or communities, and uncertainty in identifying parasite genera and/or lineages may limit the understanding of host-parasite interactions. Here, we used a multistate occupancy approach that accounts for imperfect detection to assess whether sex and breeding season influenced the prevalence of a specific *Haemoproteus* lineage (TARUF02) in a white-lined tanager population. Likewise, we explored whether the probability of detecting the target parasite in an infected bird using PCR and sequencing analyses may be influenced by season and host sex. We found little evidence that sex influenced the probability of an individual host being infected by a haemosporidian parasite. Conversely, we found that the probability of infection by *Haemoproteus* TARUF02 was ~30% higher during the breeding season, reflecting a higher prevalence of this parasite in this season. The probability that PCR detects DNA of haemosporidian parasite was higher for female birds, suggesting that they are more prone to be parasitized with parasitemia levels that are more successfully detected by molecular analysis. Sequencing successfully determined the *Haemoproteus* TARUF02 lineage in 60% of samples collected during the breeding season and 84% of samples collected during the non-breeding season. Understanding the ecology of hosts and aspects of their physiology that may influence the parasite infection is essential to better understanding of hemoparasite infections and how parasites influence their native hosts, through decreasing reproductive success, lifespan, and/or survival.

## Introduction

One of the most common parasites of wild birds are haemosporidians, vector-borne protozoans that infect several host species worldwide. Haemosporidians of the genera *Haemoproteus* are divided into two subgenera that differ in their vectors and vertebrate hosts. The subgenus *H. (Haemoproteus)* is transmitted by louse flies (Diptera: Hippoboscidae) and infects birds of the Order Columbiformes^1^ as well as seabirds of the orders Suliformes^2–4^ and Charadriiformes^4^. The subgenus *H. (Parahaemoproteus)* is transmitted primarily by *Culicoides* biting midges (Diptera: Ceratopogonidae)^1^ and infects birds of various orders and families^1^. These parasites are closely connected to their hosts with impacts ranging from sublethal effects on the fitness of the host to the decline and extinction of populations^5–8^. They can exert important selective pressure on the hosts through effects on reproductive success, lifespan, and survival^9–12^. For these reasons, avian haemosporidians are often used as a model for ecological and epidemiological studies involving investigations of host-parasite interaction dynamics, coevolution processes, and in understanding the role of parasites in the evolution of host life history^13^.

*Haemoproteus* prevalence (i.e. the proportion of the population infected in a given time period) may be influenced by a series of factors including individual features such as sex, age, and season (i.e., breeding season versus non-breeding season) and vary among different host species^14–20^. Differential exposure to vectors and host vulnerability to a particular parasite are two interacting forces that may modulate probabilities of infection among hosts and sexes^21^. For example, behaviors that differ between male and female, such as feeding habits, habitat use, or parental effort, can either enhance or reduce the probability a host is exposed to a specific parasite (e.g. ref. ^22–24^). Additionally, stress and hormonal differences between sexes may make some individuals more susceptible to a specific parasite infection. Elevated levels of testosterone during the breeding season, observed in some avian species, can be correlated with suppression of immune response to certain parasites^25^. Accordingly, birds may become more susceptible to infection during the breeding season, creating a trade-off between reproduction (i.e., parental investment) and immune defence^26^.

An important element in studies aiming to detect the prevalence of parasites in a population or community is the use of a sensitive and accurate methodology for detecting parasites. During the past few decades, the application of DNA sequencing and the definition of cytochrome b (cyt-*b*) as a molecular marker to identify parasite lineages accelerated the identification of a large diversity of haemosporidian lineages that could not be differentiated by microscopic analysis^27–29^. The use of molecular approaches, associated with a unified database of avian haemosporidian lineages^30^ allows the study of diversity, distribution, migration patterns and host specificity of these parasites and has led to increased interest in malarial infections as a model to study host-parasite systems^18,31,32^. Unfortunately, despite the high sensitivity, molecular techniques do not always guarantee success in identifying infections. The uncertainty regarding the identification of parasite genera and/or lineages infecting wild birds limits avian malaria molecular studies. The subunit of 16S rRNA is the target gene to detect infection caused by both haemosporidian parasite genera, *Plasmodium* and *Haemoproteus*, based on a standard PCR using conservative primers amplifying a broad range of parasites^33^. Thus, the parasites’ genera cannot be distinguished by PCR diagnosis, and high-quality sequencing analysis is performed to determine the parasite (i.e. *Plasmodium* or *Haemoproteus*). Due to the difficulty of producing high quality sequences for all samples in avian malaria studies^18,34–36^, those that use molecular diagnostics have a large number of positive PCR samples for which the parasite (i.e., either *Plasmodium* or *Haemoproteus*) cannot be determined. The parasitemia (i.e. the parasitic load in the host’s blood) is a crucial diagnostic component determining success in parasite identification. If an infected bird shows a very low parasitemia, the PCR may not amplify the parasite’s DNA, generating a false negative result^37,38^. Moreover, in some cases the infection can be detected, but the sequencing does not generate good quality sequences to allow the identification of infecting lineages. Other factors, including quality of extracted DNA, quality of reagents used, PCR artifacts, the appropriate PCR standardization, among others, may also influence the PCR results^39,40^.

After a period of increased number of parasites in the host’s blood (acute phase), a decrease of the parasitemia takes place in surviving specimens and the parasitemia turns into the chronic stage, when only a few parasites are found in the blood^1^. However, factors inducing a weakening of the immune system frequently lead to a short-term increase in the number of parasites in the blood (i.e. recrudescence or relapse)^41^, which occurs in most of the haemosporidian species during the breeding season of the vertebrate hosts and facilitates the infection of vectors and the transfer of infection to offspring^1^. Another factor that may interfere with haemosporidian parasitemia is the sex of the host. In vertebrates, including birds, females tend to have higher immunocompetence and be less parasitized than males^42^, which may be attributed to the direct or indirect immunosuppressive effects of testosterone in males^21,43–45^. However, some studies have shown higher parasitemia in females of different species^17,46,47^, which may be related to a number of stressors during the breeding season including spring migration, nest building, incubation and nestlings feeding. This could elevate the level of physiological stress and hence the level of chronic parasitemia in females during the breeding season.

Here, we explored a host-parasite interaction between the host white-lined tanager (*Tachyphonus rufus*, Thraupidae) and one *Haemoproteus* parasite lineage using a multistate occupancy approach^48^ that accounts for imperfect detection to yield unbiased estimates of the prevalence of the parasite lineage that infects a bird population. *Haemoproteus* genus is a good choice for investigating specific host-parasite interactions since previous avian malaria studies have postulated that *Haemoproteus* lineages are more specific to hosts than *Plasmodium* lineages^27,49–51^. We explored the influence of season and sex on the prevalence of the target parasite in a bird population. We hypothesized that parasite prevalence would be higher during the breeding season due to physiological changes that may increase host susceptibility to *Haemoproteus* infection^24^. We also evaluated whether parasite prevalence differed between males and females, which may relate to sex specific patterns of parasite susceptibility^42,52^.

We also explored whether the probability of detecting the target parasite in an infected bird using PCR and sequencing analyses may be influenced by season and sex. Specifically, we expected the parasite detection probability to vary according to the parasitemia, which might be influenced by either sex or season. Given an individual bird is infected by the target parasite, we expected a higher probability of detecting the parasite during the breeding season, when the parasitemia might be higher because of an individual’s susceptibility. Likewise, we expected different parasite detection probabilities between males and females as the parasitemia may vary according to sex specific patterns of parasite susceptibility.

Our study demonstrates how a multistate occupancy approach can be used to investigate factors influencing parasite infections in wild populations; this approach is especially important in disease ecology studies where imperfect pathogen detection can led to misleading interpretation of the parameters of interest (e.g., the prevalence of the target parasite). Likewise, because this study investigated a species-specific relationship, it was possible to observe some interesting disease ecological principles that may go unnoticed in a community context given the wide variation of effects that a parasite can have on different host species.

## Results

We captured 94 white-lined tanagers (35 females, 44 males and 15 birds for which sex identification was not possible either because they were juveniles or the field data was missing), a total of 164 times (Table S1, Supporting information). Of the 164 blood samples collected, 112 screened positive for *Plasmodium* or *Haemoproteus* and were subjected to the cytochrome *b* PCR, 71 of which successfully amplified infections and the remaining 41 positive samples were designated as uncertain. We observed the presence of multiple infections in nine of the 41 samples that failed to amplify the parasite, based on double peaks in the chromatograms; four occurrences in the breeding season (14.8% of the samples sequenced) and five in the non-breeding season (7.9% of the samples sequenced). Sequence analysis and BLAST search of the samples revealed the presence of five lineages infecting white-lined tanagers in the study site, including three *Plasmodium* lineages detected in six samples and two *Haemoproteus (Parahaemoproteus)* lineages, detected in 65 blood samples (Table S2, Supporting information). Among the positive samples for *Haemoproteus*, 64 belonged to a single lineage, TARUF02, detected here for the first time. Because the TARUF02 lineage represented almost all the *Haemoproteus* infections detected in our study (i.e., 64 out of 65 in total) and represented 90% of the amplified cytochrome *b* sequences (n = 71), dominating the haemosporidian infections in white-lined tanagers at CLBI, we defined this lineage as our target parasite in our occupancy analysis.

**Figure 1:**
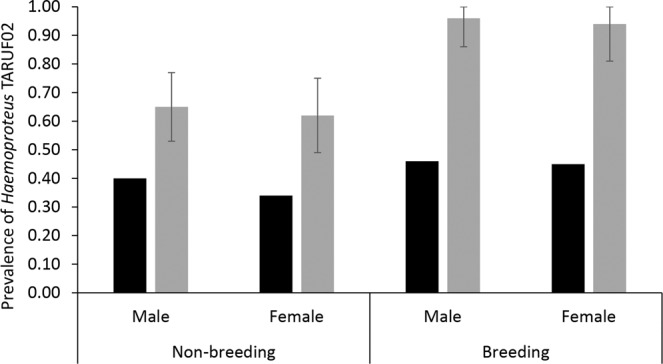
Model-averaged estimates (±SE; gray bars) of the prevalence of *Haemoproteus* TARUF02 ($\hat{\psi }$^1*2^) in male and female white-lined tanagers during the breeding and non-breeding seasons in Barreira do Inferno Rocket Launch Center of the Brazilian Air Force, Parnamirim, State of Rio Grande do Norte, Brazil. The naïve probabilities of prevalence (black bars) are given for comparison to our prevalence estimates.

We found little evidence that haemosporidian parasite infection probability (ψ^1^) varied among male and female tanagers, as the null model structure (i.e., ψ^1^ (.)) was well supported by the data (Table 1: Step 4, Supporting information Table S3). Our estimates suggest that nearly all adult tanagers had haemosporidian parasites in their blood (ψ^1^ ~ 1.0). Conversely, we found some evidence that *Haemoproteus* TARUF02 (ψ^2^) infection probability was higher during the breeding season (Table 1: Steps 3–4, Supporting information Table S3). Combining these two estimates (ψ^1^ and ψ^2^) and accounting for model selection uncertainty, the model-averaged estimates of the prevalence of *Haemoproteus* TARUF02 in white-lined tanagers (ψ^1^*^2^) are nearly identical for both sexes, but was higher during the breeding season (~0.94–0.96) when compared to the non-breeding season (~0.62–0.65; Fig. 1).

**Table 1:** Model selection results for multistate occupancy models fit to parasite detection-nondetection data from a population of white-lined tanager in Barreira do Inferno Rocket Launch Center of the Brazilian Air Force, Parnamirim, State of Rio Grande do Norte, Brazil.

Model	AICc	Delta AICc	AICc Weights	Model Likelihood	Num. Par	Deviance
**Step 1 - Modeling the probability of sequencing success (δ**_**i**,**t**_**) for an individual infected with** ***Haemoproteus*** **TARUF02**						
{ψ_i_^1^ (Season + Sex) ψ_i_^2^ (Season + Sex) p_i,t_^1^ p_i,t_^2^ (Season + Sex) δ_i,t_ (Season)}	370.25	0.00	0.63	1.00	11	346.05
{ψ_i_^1^ (Season + Sex) ψ_i_^2^ (Season + Sex) p_i,t_^1^ p_i,t_^2^ (Season + Sex) δ_i,t_ (.)}	371.88	1.63	0.28	0.44	10	350.06
**Step 2 – Modeling the probability a haemosporidan is detected in an infected individual (pi**,**t1 = pi**,**t2)**						
{ψ_i_^1^ (Season + Sex) ψ_i_^2^ (Season + Sex) p_i,t_^1^ p_i,t_^2^ (Sex) δ_i,t_ (Season)}	367.87	0.00	0.34	1.00	10	346.05
{ψ_i_^1^ (Season + Sex) ψ_i_^2^ (Season + Sex) p_i,t_^1^ p_i,t_^2^ (.) δ_i,t_ (Season)}	369.00	1.12	0.19	0.57	9	349.52
{ψ_i_^1^ (Season + Sex) ψ_i_^2^ (Season + Sex) p_i,t_^1^ p_i,t_^2^ (Sex) δ_i,t_ (.)}	369.54	1.67	0.15	0.43	9	350.06
**Step 3 - Modeling the conditional probability of being infected by Haemoproteus TARUF02 (ψi2)**, **given the individual is infected**						
{ψ_i_^1^ (Season + Sex) ψ_i_^2^ (Season) p_i,t_^1^ p_i,t_^2^ (Sex) δ_i,t_ (Season)}	365.99	0.00	0.23	1.00	9	346.51
{ψ_i_^1^ (Season + Sex) ψ_i_^2^ (.) p_i,t_^1^ p_i,t_^2^ (Sex) δ_i,t_ (.)}	366.96	0.97	0.14	0.62	7	352.06
{ψ_i_^1^ (Season + Sex) ψ_i_^2^ (Season) p_i,t_^1^ p_i,t_^2^ (.) δ_i,t_ (Season)}	367.15	1.16	0.13	0.56	8	349.98
{ψ_i_^1^ (Season + Sex) ψ_i_^2^ (Season + Sex) p_i,t_^1^ p_i,t_^2^ (Sex) δ_i,t_ (Season)}	367.87	1.88	0.09	0.39	10	346.05
**Step 4 - Modeling probability an individual is infected by a Haemosporidian (ψi1)**						
{ψ_i_^1^ (.) ψ_i_^2^ (Season) p_i,t_^1^ p_i,t_^2^ (.) δ_i,t_ (Season)}	362.65	0.00	0.20	1.00	6	349.98
{ψ_i_^1^ (.) ψ_i_^2^ (Season) p_i,t_^1^ p_i,t_^2^ (Sex) δ_i,t_ (Season)}	363.05	0.40	0.16	0.82	7	348.15
{ψ_i_^1^ (.) ψ_i_^2^ (.) p_i,t_^1^ p_i,t_^2^ (Sex) δ_i,t_ (.)}	364.17	1.52	0.09	0.47	5	353.69
{ψ_i_^1^ (Sex) ψ_i_^2^ (Season) p_i,t_^1^ p_i,t_^2^ (Sex) δ_i,t_ (Season)}	364.49	1.83	0.08	0.40	8	347.32

The most parsimonious model structures (AICc ≤ 2) for each parameter of interest were retained in subsequent steps showed below (see Supporting Information File Table S3 for complete model sets). First, the probability of *Haemoproteus* TARUF02 sequencing success (δ_i,t_) for a PCR positive sample was modeled as function of the breeding season and sex, using the general structure on other model parameters (Step 1). Retaining the best-supported δ structure, the probability of infection detection (p_i,t_^1^ = p_i,t_^2^) was modeled as function of the breeding season and sex (Step 2). Next, the conditional probability of being infected by *Haemoproteus* TARUF02 (ψ_i_^2^) was modeled as function of the breeding season and sex (Step 3). Finally, the probability of being infected by a haemosporidian (i.e. either *Plasmodium* or *Haemoproteus* - ψ_i_^1^) was modeled as function of the breeding season and sex (Step 4). The plus sign (+) indicates an additive effect between two covariates and the dot (.) indicates no covariate effects on the parameters of interest.

We also found some evidence that sex influenced the probability that haemosporidian is detected in an infected individual (**p;** Table 1: Steps 2–4, Supporting information Table S3). The probability that the PCR detects DNA of a haemosporidian parasite (either *Plasmodium* or *Haemoproteus* genera) in an infected bird was higher for females ($\hat{p}=0.74$; $\widehat{SE}=0.08$) than males ($\hat{p}=0.66$; $\widehat{SE}=0.05$). Stated differently, the probability that the PCR fails to identify haemosporidian parasite DNA is 0.34 (34%) for males and 0.26 (26%) for females.

Finally, we found evidence that sequencing success of *Haemoproteus* TARUF02 (δ) varied among seasons, and was higher during the non-breeding season ($\hat{\delta }=0.84$; $\widehat{SE}=0.11$) compared to the breeding season ($\hat{\delta }=0.60$; $\widehat{SE}=0.10$).

Combining these two estimates (**p** and δ) we can infer that given a tanager is infected with *Haemoproteus* TARUF02 during the non-breeding season, the probability of correctly identifying the *Haemoproteus* TARUF02 lineage is (0.66 * 0.84) = 0.55 (55%; $\widehat{SE}=0.08$) for males and (0.74 * 0.84) = 0.62 (62%; $\widehat{SE}=0.11$) for females. Likewise, given a tanager is infected with *Haemoproteus* TARUF02 during the breeding season, the probability of correctly identifying the *Haemoproteus* TARUF02 lineage is (0.66 * 0.60) = 0.40 (40%; $\widehat{SE}=0.07$) for males and (0.74 * 0.60) = 0.44 (44%; $\widehat{SE}=0.09$) for females.

## Discussion

Our study provides information about a specific interaction between a haemosporidian parasite and a host population, allowing us to understand relationships that might not be observed in community studies where species-specific relationships could be masked when investigating host-parasite interactions in multiple host species. Using a multistate occupancy approach, we found that: (1) nearly all tanagers were infected by haemosporidians, but the probability of infection by *Haemoproteus* TARUF02 was influenced by season; thus, (2) the prevalence of *Haemoproteus* TARUF02 was nearly identical between sexes, but higher during the breeding season; and (3) the detection of haemosporidians in infected individuals was higher in females, while the correct identification of *Haemoproteus* TARUF02 in infected individuals was higher during the non-breeding season.

The probability that the PCR test detects DNA of a haemosporidian parasite (either *Plasmodium* or *Haemoproteus* genera) from an infected bird was high (66–74%) and was slightly different between male and female tanagers. This result may indicate higher parasitemia in females, which could be caused by physiological factors including the effects of parental stress and hormones on immune function, or could imply that males are more successful at fighting the infection^21,53,54^. Unfortunately, as we did not have the blood smears from the sampled birds and cannot confirm such speculation. However, the high parasite detection in female blood suggests that a significant amount of parasite DNA is present in an aliquot of the blood sample, which may reflect a possible higher parasitemia. As the DNA extraction and PCR protocols were the same for all blood samples, we can eliminate the possibility that methodological factors, such as the quality of DNA extracted from the sample or the quality of reagents used, influenced the results obtained through PCR.

Contrary to our prediction, the probability of sequencing success for a blood sample from a tanager infected with *Haemoproteus* TARUF02 was lower during the breeding season. However, during this season we had a higher proportion of sequenced samples that indicated the presence of mixed infections (i.e., presence of double peaks in the chromatograms). This suggests that more parasite lineages may exist in the blood of the tanagers during breeding season, which would impair the efficiency of detection of our target parasite through high quality sequencing.

Our findings indicate that sequencing is quite effective for birds infected with *Haemoproteus* TARUF02 that test positive via the PCR test. These results reinforce the efficiency of using PCR-based diagnostics to detect haemosporidian infections and is an important tool for parasite-host interaction studies. However, we cannot ignore the limitations of this diagnostic approach. The most commonly used molecular techniques in haemosporidian detection do not allow the detection of parasitemia levels and often fail to identify co-infections with haemosporidian parasites that belong to the same or different genera^55,56^. For this reason, the parallel application of PCR-based techniques associated with microscopy is still considered the most effective strategy for the investigation of haemosporidians in wild hosts^57,58^. Additionally, the use of real-time PCR allows for simultaneous detection of parasites and an estimate of the level of parasitemia^59–62^, but this tool is rarely used in haemosporidian studies.

Although weak, male tanagers had higher probability of becoming infected by haemosporidian parasites (either *Plasmodium* or *Haemoproteus*), which may indicate differential exposure to vectors carrying haemosporidian parasites. Sex differences in parasite infection can be attributed to ecological or physiological factors (usually related to hormones)^21^. In our study, the slight difference observed may arise from males and females having different vector contact rates due to differences in their behavior, such as time spent foraging or unequal time spent in the nest^14,23^. If males do spend more time in areas of high vector density and/or have less time available for anti-vector behaviors when compared to females, it could increase male susceptibility to infections.

When we evaluated the conditional probability of infection by *Haemoproteus* TARUF02, we observed no differences between the sexes. Instead, we found that during the breeding season tanagers were more likely be infected with *Haemoproteus* TARUF02 than during the non-breeding season. This finding may be related to physiological and behavioral changes during the breeding season^22,26^, which are related to the fact that both reproduction and parasite defences can be costly, and during the breeding season an individual may face trade-offs between investing in offspring or in parasite defence^24^. The association between parental investment and haemosporidian prevalence has been shown in various bird species and demonstrated that reproductive effort increases susceptibility to haematozoan infection^14,24,26,63^. The probability of infection by *Haemoproteus* TARUF02 may also be related to vector abundance, since seasonal climatic variations influence temperature and rainfall, which affect vector reproduction^64^. The temperature also affects the development of the parasites in the vectors and the frequency of blood feeding^64,65^. Unfortunately, we lack data on the diversity and abundance of *Haemoproteus* vectors in CLBI. To better understand the transmission dynamics of *Haemoproteus* TARUF02 in white-lined tanagers, future studies should identify the biting midges acting as vectors of this parasite in CLBI. The lack of information about avian haemosporidian vectors remains a challenge and limits studies of avian malaria infection dynamics in wild birds, and requires more attention in future studies.

The prevalence of *Haemoproteus* TARUF02 in the white-lined tanager population (62–96%) was higher than the prevalence observed in studies of haemosporidian infections in other wild bird populations (e.g. refs. ^49,66–68^) and was higher during the breeding season. This lineage dominates the haemosporidian infections in white-lined tanagers at CLBI, a relationship that deserves attention as *Haemoproteus* TARUF02 likely has certain specificity for this host species and an advantage in infecting it when competing with other lineages.

Our study focused on a specific parasite-host interaction in the natural environment, which is still rare in literature. We found higher infectivity rates and prevalence of *Haemoproteus* TAFUR02 during the breeding season, which could influence reproductive success of infected individuals. Our data also suggests that infected females are more heavily parasitized, having infections that are more detectable by molecular analysis. Parasite detection in individuals with a low parasitic load is not perfect and naïve prevalence values likely underestimate true prevalence in the bird population or community. Thus, understanding the ecology of vectors and hosts and aspects of their physiology that may influence the parasite load should be of fundamental importance to ensure greater accuracy in the evaluation of hemoparasite infections through molecular analysis. Studies focusing on host-parasite interactions using an entire community of organisms may fail to reveal host-specific differences in parasite infections.

## Material and Methods

### Study site and Host species

The white-lined tanager (*Tachyphonus rufus*) is a passerine bird endemic to the Neotropics and very common in the northeastern coast of Brazil. The species feeds on fruits and insects and forages in the lower and middle strata of vegetation^69,70^. Similar-sized male and female adults (average 34.4 g) are typically found in pairs, but are sexually dimorphic allowing for easy discrimination in the field. In our study site, the breeding season extends from February to May^71^ and we used this information to evaluate seasonal differences in prevalence and detection probability of the parasite.

**Figure 2:**
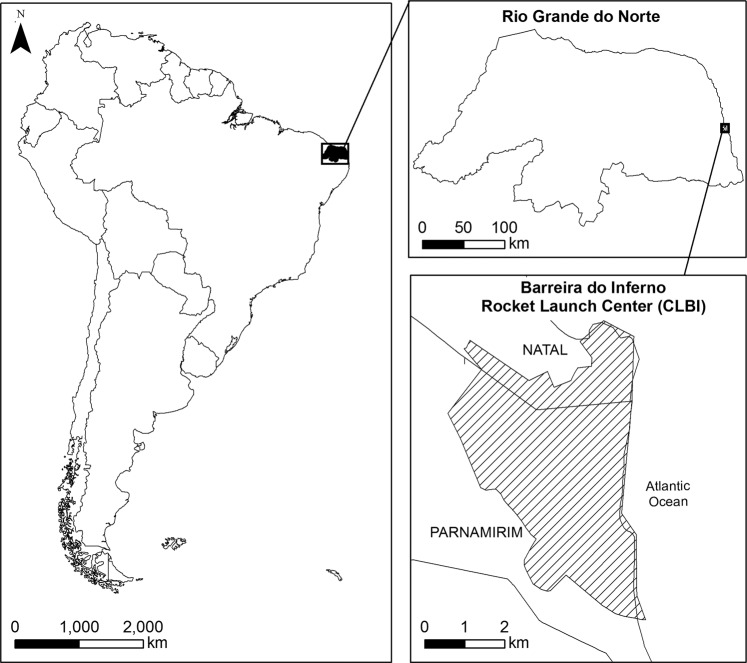
Location of the study site sampled for a white-lined tanager population infected by *Haemoproteus* TARUF02, Barreira do Inferno Rocket Launch Center of the Brazilian Air Force, Parnamirim, State of Rio Grande do Norte, Brazil.

The study was carried out in Barreira do Inferno Rocket Launch Center of the Brazilian Air Force (Centro de Lançamento Barreira do Inferno – CLBI), in Parnamirim, State of Rio Grande do Norte (Fig. 2). This restricted access area has no signs of wood removal, hunting, or burning. The 1,800 ha study area is located in a tropical coastal vegetation region, named *Restinga*, which is a type of vegetation of the Atlantic Forest domain. The soil is sandy and covered by xerophytic vegetation, with a predominance of herbaceous plants and shrubs, and is classified as Lowland Semideciduous Forest. The climate is classified as tropical, with dry summers and rainy winters^72^. The mean temperature was 26.5 °C and the total precipitation was 1,971.5 mm per year during the study period (Fig. S1, Supporting information). Thus, the rainy season was defined as the period from March to September and the dry season spans the period from October to February.

### Bird sampling

Our use of mist-nets and banding at the fieldwork was approved by the Brazilian biodiversity monitoring agency (Institute Chico Mendes for Biodiversity Conservation—ICMBio, Brazilian National Center for Bird Conservation—CEMAVE, permission 3239). We followed standard ethical protocols for wildlife animals. Time in captivity was kept to a minimum, and all individuals were released at the same place they were captured. This study was approved by the Ethics Committee in Animal Experimentation (CETEA), Universidade Federal de Minas Gerais, Brazil (Protocol #254/2011).

We studied host-disease dynamics in a white-lined tanager population within a 30 ha plot (550 m × 550 m) from April 2013 to December 2014. We used a grid of 11 × 11 lines spaced at 50 m intervals to establish 121 mist-net locations at the intersection of the grid lines. We sampled 36–49 of these points each month in a cyclical way so that all points were sampled once every three month. Captures were made using mist-nets (Ecotone 18 × 3 m, five shelves and mesh 19 mm), opened at 4:50 a.m. and closed 9:30 a.m. Nets were checked every 60 minutes and captured birds were identified to species, sexed (according to plumage), and tagged with individual aluminum bands provided by the Brazilian National Center for Bird Conservation (CEMAVE/ICMBio). We collected blood samples of all individuals on filter paper via brachial venipuncture with a sterile needle (13 × 4.5 mm); samples were stored at 4 °C until DNA extraction. Blood samples were collected for all birds (both initial and recaptured individuals) to screen for parasites during each capture occasion.

### Parasite detection

DNA was extracted from avian blood samples using the phenol-chloroform method followed by isopropanol precipitation^73^. The genomic DNA pellet was resuspended in 35 µL of TE 1 × (10 mM Tris-HCl, pH 7.4; 1 mM EDTA, pH 8.0) and quantified using a NanoDrop. DNA samples were initially screened for the presence of *Plasmodium*/*Haemoproteus* infections by PCR using the primers 343 F (5′GCTCACGCATCGCTTCT3′) and 496 R (5′GACCGGTCATTTTCTTTG3′) described by Fallon *et al*.^33^. PCR products were viewed on a 6% acrylamide gel. Subsequent to parasite screening, a 524 bp fragment of the mtDNA cytochrome *b* gene from the infected individuals was amplified by a nested-PCR. For the first amplification we used the primers HaemNFI (5′CATATATTAAGAGAAITATGGAG3′) and HaemNR3 (5′ATAGAAAGATAAGAAATACCATTC3′); and for the second amplification the primers were HaemF (5′ATGGTGCTTTCGATATATGCATG3′) and HaemR2 (5′GCATTATCTGGATGTGATAATGGT3′), following protocols described by Hellgren *et al*.^28^. Positive and negative controls were used in each PCR batch. The positive controls consisted of DNA extracted from blood samples of chickens experimentally infected with *Plasmodium gallinaceum*, and the negative controls were ultrapure water.

PCR products were purified using a solution of 20% polyethylene-glycol 8,000 according to the methods of Sambrook and Russell^73^ with modifications. After purification, PCR products were sequenced in both directions using the BigDye Terminator Kit v3 (Applied Biosystems, Foster City, CA, USA) using an ABI3100H automated sequencer (Applied Biosystems, Foster City, CA, USA). DNA sequences were aligned and edited using ChromasPro version 2.0.1 (Technelysium Pty Ltd) and compared with data available in the open access databases Genbank and MalAvi^30^. Samples containing differences in one or more nucleotides were considered as distinct cytochrome *b* lineages. Genetic lineages of parasites represent independent evolutionary entities that do not have recombination between them, and thus can be considered as distinct biological species^74,75^. A genetic sequence is the result obtained after the sequencing of the parasite infecting each individual bird, so the same lineage can be detected in several birds of the same or different species.

Lineages without previous records in the database were considered new lineages and deposited in GenBank under accession number MH260577. New occurrences of sequences previously described were also deposited in GenBank under accession numbers MH341735, MH341736, MH341737, and MH341738.

### Modeling model parameters as function of predictor covariates

We used the single-season multistate occupancy model^48^ available in Program MARK^76^ which allows three observed states in the capture histories of individual birds. When an individual bird (i.e., our sample unit or ‘site’) was captured in a specific monthly survey (i.e., sampling occasion) and the tests were negative for the target parasite (i.e., no haemosporidian – neither the *Plasmodium* or *Haemoproteus* parasite was detected in the individual blood sample) the observed state = 0. If the tests were positive for the target parasite (i.e., the individual blood sample was infected with a haemosporidian parasite, specifically *Haemoproteus* TARUF02 lineage) the observed state = 2. When an individual bird was captured, but the tests were ambiguous (i.e., the blood sample was positive on PCR screening but the parasite was not identified or the parasite was identified but was not *Haemoproteus* TARUF02 lineage), it was defined as our uncertain state (observed state = 1).

Using the observed data for each individual captured in each breeding or non-breeding season, we estimated the following model parameters:

**ψ**_**i**_^**1**^ = probability that a captured individual *i* was infected with haemosporidian, regardless of the lineage (*Plasmodium* or *Haemoproteus* parasite; true state = 1 or 2);

**ψ**_**i**_^**2**^ = probability that a captured individual *i* was infected with *Haemoproteus* TARUF02, given that the individual was infected with a haemosporidian (true state = 2 | true state = 1 or 2);

Note, the prevalence of *Haemoproteus* TARUF02 in the bird population is obtained via the product: **ψ**_**i**_^1*2^ = **ψ**_**i**_^**1**^ **ψ**_**i**_^**2**^.

**p**_**i**,**t**_^**1**^ = probability that haemosporidian was detected via PCR for individual *i*, during occasion *t*, given the individual was infected with a parasite that was not the *Haemoproteus* TARUF02 lineage (Probability (haemosporidian detected | true state = 1));

**p**_**i**,**t**_^**2**^ = probability that haemosporidian was detected via PCR for individual *i*, during occasion *t*, given the individual was infected with the *Haemoproteus* TARUF02 lineage (Probability (haemosporidian detected| true state = 2));

**δ**_**i**,**t**_ = probability that *Haemoproteus* TARUF02 was identified via sequencing, given that haemosporidian were detected (via PCR) for infected individual *i*, during occasion *t* (Probability (observed state 2|true state = 2 and haemosporidian were detected via PCR)).

We modeled the above parameters as a function of season and/or sex. Our sampling occurred over two breeding seasons (April-May 2013 (2 monthly occasions); Feb-May 2014 (4 monthly occasions)) and two non-breeding seasons (June 2013-Jan 2014 (8 monthly occasions); June-Dec 2014 (7 monthly occasions)). We assume that prevalence was similar among the two breeding and two non-breeding seasons (i.e., we did not consider year effects). We only included adult individuals for which sex was known in our occupancy analysis. A subset of individuals were captured during more than one breeding/non-breeding season (Table S1, Supporting information), but we consider these individuals to be a representative sample of the population of tanagers using the study area during a given season. We had no reason to believe that parasite lineage influenced the detection of haemosporidan via PCR, so we assume **p**_**i**,**t**_^**1**^ = **p**_**i**,**t**_^**2**^ = **p**_**i**,**t**_ and modeled these parameters using a shared intercept. We used the Akaike Information Criterion, adjusted for small sample sizes (AICc), to determine which of our competing models/hypotheses, and associated covariate(s), were most parsimonious^77^. We adopted a “step-down”^78^ strategy to evaluate our candidate models. Using the most parameterized model structure containing all the covariates that included sex and seasonal effects for parasite occurrence states (ψ^1^, ψ^2^) and PCR detection probability (**p**), we first explored the effects of these same covariates on *Haemoproteus* TARUF02 sequencing success (δ; Table 1: Step 1, Supporting information Table S3). Retaining the best-supported model structures for *Haemoproteus* TARUF02 sequencing success (δ models with Δ AICc ≤ 2), we explored individual and additive effects of breeding season and sex on the probability haemosporidan is detected in an infected individual (**p;** Table 1: Step 2, Supporting information Table S3). The same strategy was adopted to identify the most explanatory covariates for parasite occupancy states, ψ^1^ and ψ^2^ (Table 1: Steps 3–4, Supporting information Table S3). This process enabled us to identify the covariates that influenced the parameters of interest for the target bird population. We used the maximum likelihood procedures available in Program MARK to estimate the parameters of interest. Because of model selection uncertainty, we report model-averaged estimates of model parameters^77^.

## Supplementary information

Supplementary Information File.

## Data Availability

The datasets generated during and/or analysed during the current study are available in the Supplementary Information files, further data are available from the corresponding author on reasonable request.
