# Supplementary material for: Using a multistate occupancy approach to determine molecular diagnostic accuracy and factors affecting avian haemosporidian infections
Source: Sci Rep. 2020 May 21;10:8480. doi: 10.1038/s41598-020-65523-x (PMC7242334; doi:10.1038/s41598-020-65523-x)
Supplement: Supplementary file 1 — Supplementary Information File. [file 41598_2020_65523_MOESM1_ESM.pdf]

Supplementary Information File

Using a multistate occupancy approach to determine molecular diagnostic accuracy and factors affecting avian haemosporidian infections

Raquel A. Rodrigues, Rodrigo L. Massara, Larissa L. Bailey, Mauro Pichorim, Patrícia A. Moreira, Érika M. Braga

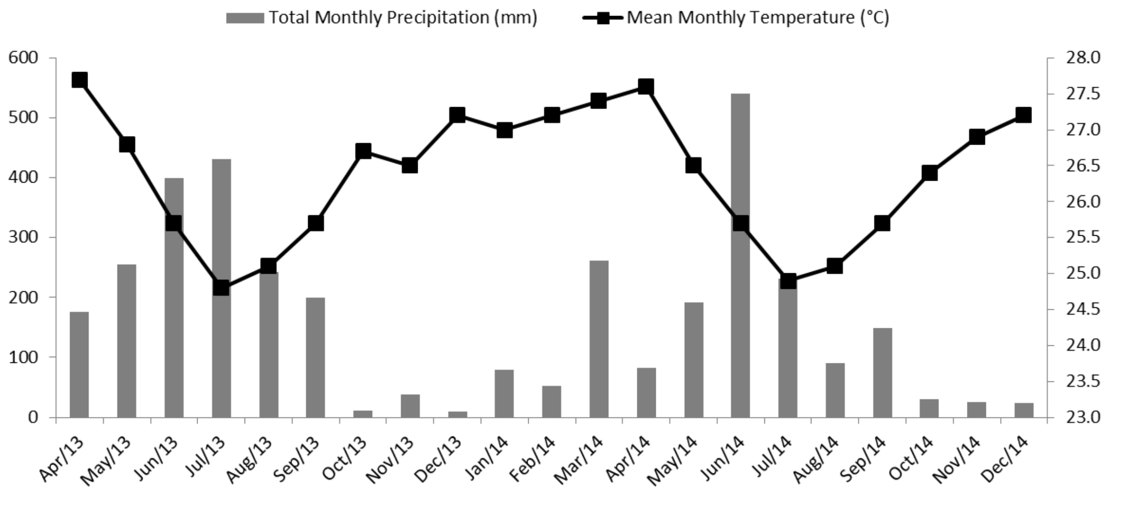

Fig. S1. Climatogram representing the average monthly temperatures and total monthly precipitation during the period of study in Barreira do Inferno Rocket Launch Center of the Brazilian Air Force, Parnamirim, State of Rio Grande do Norte, Brazil.

Table S1. White-lined tanager individuals captured over 21 months of study (April 2013 to December 2014) and their infection status by *Plasmodium* or *Haemoproteus* detected from sequencing of mitochondrial cytochrome *b* gene. C = individual first catch; R1-R8 = recaptures; M/Y= Month/Year when the bird was captured; P = positive diagnostic without lineage identification; N = negative diagnostic; ND = positive in a previous capture, but not detected in the current event. TARUF02 = *Haemoproteus* (*Parahaemoproteus*) TARUF02 lineage; PAMIT01 = *Plasmodium* PAMIT01 lineage; PADOM11 = *Plasmodium* PADOM11 lineage; UN203 = *Haemoproteus* (*Parahaemoproteus*) UN203 lineage; BAFLA04 = *Plasmodium* BAFLA04 lineage.

	**Recaptures**																	
	**C**		**R1**		**R2**		**R3**		**R4**		**R5**		**R6**		**R7**		**R8**	
Individual	M/Y	Diagnostic	M/Y	Diagnostic	M/Y	Diagnostic	M/Y	Diagnostic	M/Y	Diagnostic	M/Y	Diagnostic	M/Y	Diagnostic	M/Y	Diagnostic	M/Y	Diagnostic
1	jul/13	TARUF02	sep/13	TARUF02														
2	jul/13	N	dec/14	N														
3	sep/13	N	mar/14	N														
4	sep/13	TARUF02	feb/14	TARUF02														
5	sep/13	TARUF02	mar/14	ND														
6	jun/13	TARUF02	sep/13	TARUF02														
7	aug/13	TARUF02	sep/13	TARUF02	dec/13	TARUF02	feb/14	PAMIT01	mar/14	TARUF02								
8	may/13	P	feb/14	P	apr/14	TARUF02												
9	aug/13	TARUF02	feb/14	TARUF02														
10	nov/13	N	dec/13	TARUF02														
11	jan/14	TARUF02	feb/14	TARUF02	dec/14	P												
12	jun/14	P	nov/14	P														
13	dec/13	N	may/14	P	jun/14	N	sep/14	P	oct/14	N								
14	dec/13	PADOM11	jan/14	TARUF02	feb/14	TARUF02	apr/14	P	may/14	ND	aug/14	TARUF02						
15	jan/14	TARUF02	mar/14	TARUF02	may/14	P	aug/14	P	nov/14	P								
16	jan/14	TARUF02	oct/14	P														
17	oct/14	P	nov/14	N														
18	sep/14	P	nov/14	P														

19	apr/13	N	dec/13	UN203														
20	may/13	N	oct/13	TARUF02														
21	jun/13	TARUF02	oct/13	TARUF02														
22	jun/13	TARUF02	jul/13	TARUF02	aug/13	TARUF02	oct/13	TARUF02	nov/13	P	dec/13	TARUF02	feb/14	TARUF02	apr/14	P	nov/14	TARUF02
23	sep/13	P	mar/14	BAFLA04	jul/14	BAFLA04	sep/14	ND										
24	oct/13	TARUF02	apr/14	ND	jul/14	P												
25	mar/14	TARUF02	sep/14	N														
26	may/13	TARUF02	dec/13	ND	feb/14	TARUF02	mar/14	ND	sep/14	TARUF02								
27	may/13	TARUF02	jan/14	TARUF02	aug/14	ND	sep/14	TARUF02										
28	dec/13	N	aug/14	N														
29	may/13	N	nov/13	TARUF02	feb/14	TARUF02	may/14	ND	sep/14	P								
30	sep/13	N	dec/13	P	jun/14	BAFLA04												
31	aug/13	N	sep/13	TARUF02														
32	nov/13	TARUF02	jan/14	TARUF02														
33	dec/13	N	jan/14	TARUF02	mar/14	TARUF02	sep/14	P										
34	apr/13	N																
35	mar/14	TARUF02																
36	jun/14	N																
37	sep/13	TARUF02																
38	sep/13	TARUF02																
39	nov/14	N																
40	dec/14	P																
41	jun/13	P																
42	mar/14	P																

43	jun/14	N							
44	oct/14	N							
45	sep/13	N							
46	dec/14	N							
47	oct/13	TARUF02							
48	oct/13	TARUF02							
49	nov/13	P							
50	dec/13	P							
51	dec/13	P							
52	mar/14	N							
53	mar/14	TARUF02							
54	mar/14	P							
55	mar/14	N							
56	oct/14	N							
57	apr/14	P							
58	may/14	N							
59	may/14	N							
60	jan/14	P							
61	jan/14	P							
62	jan/14	TARUF02							
63	feb/14	N							

64	jul/14	N							
65	aug/14	N							
66	nov/14	N							
67	nov/14	N							
68	nov/14	P							
69	oct/14	P							
70	nov/14	TARUF02							
71	sep/14	N							
72	dec/13	TARUF02							
73	oct/13	N							
74	may/13	TARUF02							
75	feb/14	P							
76	feb/14	P							
77	sep/14	TARUF02							
78	feb/14	P							
79	dec/13	P							
80	oct/13	N							
81	oct/13	N							
82	jul/14	N							
83	sep/13	N							
84	jul/13	TARUF02							

85	nov/13	TARUF02								
86	aug/13	N								
87	dec/14	TARUF02								
88	dec/14	P								
89	dec/14	N								
90	dec/14	N								
91	dec/14	TARUF02								
92	dec/14	N								
93	dec/14	TARUF02								
94	dec/14	P								

Table S2. Haemosporidian lineages found in white-lined tanager *Tachyphonus rufus* individuals sampled in Barreira do Inferno Rocket Launch Center of the Brazilian Air Force, Parnamirim, State of Rio Grande do Norte, Brazil. S= number of samples in which this lineage was detected (including individual recaptures); N = number of infected individuals

**Lineage**	**Parasite genera**	**S**	**N**	**Genbank**
BAFLA04	*Plasmodium*	04	03	MH341735
PADOM11	*Plasmodium*	01	01	MH341736
PAMIT01	*Plasmodium*	01	01	MH341737
TARUF02	*Haemoproteus (Parahaemoproteus)*	64	32	MH260577
UN203	*Haemoproteus (Parahaemoproteus)*	01	01	MH341738

Table S3. Model selection results for multistate occupancy models fit to parasite detection-nondetection data from a population of white-lined tanager in Barreira do Inferno Rocket Launch Center of the Brazilian Air Force, Parnamirim, State of Rio Grande do Norte, Brazil. The most parsimonious model structures (AICc ≤ 2) for each parameter of interest were retained in subsequent steps showed below. First, the probability of *Haemoproteus* TARUF02 sequencing success ($\delta_{i,t}$) for a PCR positive sample was modeled as function of the breeding season and sex, using the general structure on other model parameters (Step 1). Retaining the best-supported δ structure, the probability of infection detection ($p_{i,t}^1 = p_{i,t}^2$) was modeled as function of the breeding season and sex (Step 2). Next, the conditional probability of being infected by *Haemoproteus* TARUF02 (ψ_i^2) was modeled as function of the breeding season and sex (Step 3). Finally, the probability of being infected by a haemosporidian (i.e. either *Plasmodium* or *Haemoproteus* - ψ_i^1) was modeled as function of the breeding season and sex (Step 4). The plus sign (+) indicates an additive effect between two covariates and the dot (.) indicates no covariate effects on the parameters of interest.

Step 1 - Modeling the probability of sequencing success ($\delta_{i,t}$) for an individual infected with *Haemoproteus* TARUF02

Model	AICc	Delta AICc	AICc Weights	Model Likelihood	Num. Par	Deviance
{ψ_i^1 (Season + Sex) ψ_i^2 (Season + Sex) $p_{i,t}^1$ $p_{i,t}^2$ (Season + Sex) $\delta_{i,t}$ (Season)}	370.25	0.00	0.63	1.00	11	346.05
{ψ_i^1 (Season + Sex) ψ_i^2 (Season + Sex) $p_{i,t}^1$ $p_{i,t}^2$ (Season + Sex) $\delta_{i,t}$ (.)}	371.88	1.63	0.28	0.44	10	350.06
{ψ_i^1 (Season + Sex) ψ_i^2 (Season + Sex) $p_{i,t}^1$ $p_{i,t}^2$ (Season + Sex) $\delta_{i,t}$ (Sex)}	374.23	3.98	0.09	0.14	11	350.03

Step 2 – Modeling the probability a haemosporidan is detected in an infected individual ($p_{i,t}^1 = p_{i,t}^2$)

Model	AICc	Delta AICc	AICc Weights	Model Likelihood	Num. Par	Deviance
{ψ_i^1 (Season + Sex) ψ_i^2 (Season + Sex) $p_{i,t}^1$ $p_{i,t}^2$ (Sex) $\delta_{i,t}$ (Season)}	367.87	0.00	0.34	1.00	10	346.05
{ψ_i^1 (Season + Sex) ψ_i^2 (Season + Sex) $p_{i,t}^1$ $p_{i,t}^2$ (.) $\delta_{i,t}$ (Season)}	369.00	1.12	0.19	0.57	9	349.52
{ψ_i^1 (Season + Sex) ψ_i^2 (Season + Sex) $p_{i,t}^1$ $p_{i,t}^2$ (Sex) $\delta_{i,t}$ (.)}	369.54	1.67	0.15	0.43	9	350.06
{ψ_i^1 (Season + Sex) ψ_i^2 (Season + Sex) $p_{i,t}^1$ $p_{i,t}^2$ (Season + Sex) $\delta_{i,t}$ (Season)}	370.25	2.38	0.10	0.30	11	346.05
{ψ_i^1 (Season + Sex) ψ_i^2 (Season + Sex) $p_{i,t}^1$ $p_{i,t}^2$ (.) $\delta_{i,t}$ (.)}	370.70	2.83	0.08	0.24	8	353.53
{ψ_i^1 (Season + Sex) ψ_i^2 (Season + Sex) $p_{i,t}^1$ $p_{i,t}^2$ (Season) $\delta_{i,t}$ (Season)}	371.04	3.17	0.07	0.21	10	349.22
{ψ_i^1 (Season + Sex) ψ_i^2 (Season + Sex) $p_{i,t}^1$ $p_{i,t}^2$ (Season + Sex) $\delta_{i,t}$ (.)}	371.88	4.01	0.05	0.14	10	350.06
{ψ_i^1 (Season + Sex) ψ_i^2 (Season + Sex) $p_{i,t}^1$ $p_{i,t}^2$ (Season) $\delta_{i,t}$ (.)}	372.71	4.83	0.03	0.09	9	353.23

Step 3 - Modeling the conditional probability of being infected by *Haemoproteus* TARUF02 (ψ_i^2), given the individual is infected

Model	AICc	Delta AICc	AICc Weights	Model Likelihood	Num. Par	Deviance
{ψ_i^1 (Season + Sex) ψ_i^2 (Season) $p_{i,t}^1$ $p_{i,t}^2$ (Sex) $\delta_{i,t}$ (Season)}	365.99	0.00	0.23	1.00	9	346.51
{ψ_i^1 (Season + Sex) ψ_i^2 (.) $p_{i,t}^1$ $p_{i,t}^2$ (Sex) $\delta_{i,t}$ (.)}	366.96	0.97	0.14	0.62	7	352.06
{ψ_i^1 (Season + Sex) ψ_i^2 (Season) $p_{i,t}^1$ $p_{i,t}^2$ (.) $\delta_{i,t}$ (Season)}	367.15	1.16	0.13	0.56	8	349.98
{ψ_i^1 (Season + Sex) ψ_i^2 (Season + Sex) $p_{i,t}^1$ $p_{i,t}^2$ (Sex) $\delta_{i,t}$ (Season)}	367.87	1.88	0.09	0.39	10	346.05
{ψ_i^1 (Season + Sex) ψ_i^2 (Sex) $p_{i,t}^1$ $p_{i,t}^2$ (Sex) $\delta_{i,t}$ (.)}	368.07	2.08	0.08	0.35	8	350.90
{ψ_i^1 (Season + Sex) ψ_i^2 (Season) $p_{i,t}^1$ $p_{i,t}^2$ (Sex) $\delta_{i,t}$ (.)}	368.08	2.09	0.08	0.35	8	350.90
{ψ_i^1 (Season + Sex) ψ_i^2 (.) $p_{i,t}^1$ $p_{i,t}^2$ (Sex) $\delta_{i,t}$ (Season)}	368.75	2.76	0.06	0.25	8	351.58
{ψ_i^1 (Season + Sex) ψ_i^2 (Season + Sex) $p_{i,t}^1$ $p_{i,t}^2$ (.) $\delta_{i,t}$ (Season)}	369.00	3.01	0.05	0.22	9	349.52

{ψ_i^1 (Season + Sex) ψ_i^2 (Season + Sex) $p_{i,t}^1$ $p_{i,t}^2$ (Sex) $\delta_{i,t}$ (.)}	369.54	3.55	0.04	0.17	9	350.06
{ψ_i^1 (Season + Sex) ψ_i^2 (Sex) $p_{i,t}^1$ $p_{i,t}^2$ (Sex) $\delta_{i,t}$ (Season)}	369.91	3.92	0.03	0.14	9	350.44
{ψ_i^1 (Season + Sex) ψ_i^2 (.) $p_{i,t}^1$ $p_{i,t}^2$ (.) $\delta_{i,t}$ (Season)}	369.95	3.96	0.03	0.14	7	355.05
{ψ_i^1 (Season + Sex) ψ_i^2 (Sex) $p_{i,t}^1$ $p_{i,t}^2$ (.) $\delta_{i,t}$ (Season)}	371.07	5.08	0.02	0.08	8	353.90

Step 4 - Modeling probability an individual is infected by a Haemosporidian (ψ_i^1)

Model	AICc	Delta AICc	AICc Weights	Model Likelihood	Num. Par	Deviance
{ψ_i^1 (.) ψ_i^2 (Season) $p_{i,t}^1$ $p_{i,t}^2$ (.) $\delta_{i,t}$ (Season)}	362.65	0.00	0.20	1.00	6	349.98
{ψ_i^1 (.) ψ_i^2 (Season) $p_{i,t}^1$ $p_{i,t}^2$ (Sex) $\delta_{i,t}$ (Season)}	363.05	0.40	0.16	0.82	7	348.15
{ψ_i^1 (.) ψ_i^2 (.) $p_{i,t}^1$ $p_{i,t}^2$ (Sex) $\delta_{i,t}$ (.)}	364.17	1.52	0.09	0.47	5	353.69
{ψ_i^1 (Sex) ψ_i^2 (Season) $p_{i,t}^1$ $p_{i,t}^2$ (Sex) $\delta_{i,t}$ (Season)}	364.49	1.83	0.08	0.40	8	347.32
{ψ_i^1 (.) ψ_i^2 (Season + Sex) $p_{i,t}^1$ $p_{i,t}^2$ (Sex) $\delta_{i,t}$ (Season)}	364.86	2.21	0.07	0.33	8	347.69
{ψ_i^1 (Season)ψ_i^2 (Season) $p_{i,t}^1$ $p_{i,t}^2$ (.) $\delta_{i,t}$ (Season)}	364.88	2.23	0.06	0.33	7	349.98
{ψ_i^1 (Sex) ψ_i^2 (Season) $p_{i,t}^1$ $p_{i,t}^2$ (.) $\delta_{i,t}$ (Season)}	364.88	2.23	0.06	0.33	7	349.98
{ψ_i^1 (Season) ψ_i^2 (Season) $p_{i,t}^1$ $p_{i,t}^2$ (Sex) $\delta_{i,t}$ (Season)}	365.30	2.65	0.05	0.27	8	348.13
{ψ_i^1 (Sex) ψ_i^2 (.) $p_{i,t}^1$ $p_{i,t}^2$ (Sex) $\delta_{i,t}$ (.)}	365.53	2.88	0.05	0.24	6	352.86
{ψ_i^1 (Season + Sex) ψ_i^2 (Season) $p_{i,t}^1$ $p_{i,t}^2$ (Sex) $\delta_{i,t}$ (Season)}	365.99	3.34	0.04	0.19	9	346.51
{ψ_i^1 (Sex) ψ_i^2 (Season + Sex) $p_{i,t}^1$ $p_{i,t}^2$ (Sex) $\delta_{i,t}$ (Season)}	366.33	3.68	0.03	0.16	9	346.86
{ψ_i^1 (Season) ψ_i^2 (.) $p_{i,t}^1$ $p_{i,t}^2$ (Sex) $\delta_{i,t}$ (.)}	366.35	3.70	0.03	0.16	6	353.68
{ψ_i^1 (Season + Sex) ψ_i^2 (.) $p_{i,t}^1$ $p_{i,t}^2$ (Sex) $\delta_{i,t}$ (.)}	366.96	4.31	0.02	0.12	7	352.06
{ψ_i^1 (Season) ψ_i^2 (Season + Sex) $p_{i,t}^1$ $p_{i,t}^2$ (Sex) $\delta_{i,t}$ (Season)}	367.15	4.49	0.02	0.11	9	347.67
{ψ_i^1 (Season + Sex) ψ_i^2 (Season) $p_{i,t}^1$ $p_{i,t}^2$ (.) $\delta_{i,t}$ (Season)}	367.15	4.50	0.02	0.11	8	349.98
{ψ_i^1 (Season + Sex) ψ_i^2 (Season + Sex) $p_{i,t}^1$ $p_{i,t}^2$ (Sex) $\delta_{i,t}$ (Season)}	367.87	5.22	0.01	0.07	10	346.05
